# Commodities and cognition

**DOI:** 10.3389/fpsyg.2014.01181

**Published:** 2014-11-04

**Authors:** Paul Loader

**Affiliations:** Informatics, University of SussexBrighton, UK

**Keywords:** Sohn-Rethel, cognition, commodities, apriorism, empiricism

John Stewart's paper examines arguments for a social explanation of the conceptual categories which Kant and others have posited as the pre-requisite for abstract cognition. Such an explanation would have the advantage of providing a satisfactory alternative to both empiricism, which cannot tell us how we get from the particular to the universal, and Kantian apriorism which does not really offer an explanation (p. 2).

Stewart looks first at Durkheim's (1915) account which, focusing on ethnographic studies of Australian aborigines, argues that abstract concepts used in these societies had their origins in (essentially social) religious categories. Stewart spends the bulk of the paper, however, examining Sohn-Rethel's (1978) account. Combining Marx's twin insights that ideas are “sublimates” of the “material life process” (Marx, 1844, p. 104) and that the “mystical character” of the commodity is such that “not an atom of matter” (Marx, 1867, p. 138) enters into its existence as an exchange value, Sohn-Rethel argues that the “enigmatic cognitive faculties of civilized man” (1978, p. 34) have their roots in commodity exchange. In particular abstract non-empirical concepts are derived ultimately from the “real abstraction” of commodity exchange. Stewart selects some of Sohn-Rethel's own “homologies” to demonstrate this relation: solipsism corresponds to the situation of the agent in exchange wherein “the action is social, the mind is private” (p. 43); Parmenidean “oneness” derives from the universal equivalence between commodities expressed through exchange value; “abstract quantity” from the “non-dimensional quantity” which the act of exchange attributes to objects as commodities; “abstract time” from the immutability of the object considered as commodity, and so on.

Stewart makes a good case for Sohn-Rethel as presenting an account of cognition which is “social” in a more thoroughgoing way than is typically countenanced by embodied, extended, and distributed approaches. Moreover in highlighting Sohn-Rethel's work within this context, Stewart's paper may serve to generate discussion in other, equally illuminating, directions. In particular we might note Sohn-Rethel's rejection of the traditional view that “abstraction is the inherent activity and the exclusive privilege of thought” (p. 19), in favor of a conception whereby abstraction initially manifests itself through action. With “real abstraction,” says Sohn-Rethel, “only the action is abstract, the consciousness of the actors is not” (p. 30). Thus, we would seem to have an instance of a higher form of cognition constituting itself enactively (and collectively). This perhaps provides the germs for an interesting challenge to accounts which, whilst embracing various elements of an embodied, embedded, outlook, still reserve a non-enactive space for |offline cognition,” with the latter characterized solely in terms of the inner representational states of individual agents, decoupled from real-time interaction with the world (e.g., Clark and Grush, 1999; Wheeler, 2005).

We might add that Stewart is to be commended for introducing an unambiguously Marxian theorist into the arena of cognitive science. Marx has had some significant influence on recent currents in cognitive science, via intermediaries such as Vygotsky, Merleau-Ponty, and Levins and Lewontin but the Marxian character of this influence is rarely acknowledged. Here the relevance of at least one strand of Marxian theory is made explicit.

Insofar as there may be difficulties with aspects of Stewart's account, these perhaps have less to do with Stewart's own admirably lucid summary and analysis of Sohn-Rethel's book, and more to do with details of the latter's own argument. It might be suggested, for example, that “solipsism” is not really a conceptual category on a par with “time,” “space,” “oneness” etc. but is rather a kind of philosophical aberration (albeit one that may well have its roots in the alienation of commodity exchange.) Connectedly, some of Sohn-Rethel's arguments for a connection between particular conceptual categories and the exchange nexus seem more convincing than others, a fact which perhaps leaves the reader wishing for an independent criterion by means of which the correctness or otherwise of these correlations can be assessed. There is also, for this reader, at least, an apparent equivocation (in Sohn-Rethel's account) between the idea that commodity exchange is the source of conceptual abstraction *per se* and the idea that commodity exchange is the source of particular abstract concepts. None of these points, however, should be seen as detracting from the originality and overall plausibility of Sohn-Rethel's position.

If there is one area of tension within Stewart's own analysis this is perhaps to be found in his concern with the precise causal relation between social forms and conceptual forms. He argues that “the social forms and the thought forms come about together; while there is a sense in which it is the social forms which provide the ground for the conceptual forms… it is surely at least as much the other way round: the cognitive capacity to think in a certain way is a condition for the corresponding form of social life to arise.” (p. 6) Stewart's predicament here is, in part, a familiar one, recognizable both to dynamically oriented cognitive scientists, and to dialectically oriented Marxists alike. It is the problem of how to elucidate reciprocal causal relations in any particular instance without appearing to give ground to either side of the causal equation in isolation. In this case, conceding that “a cognitive capacity to think in a certain way” is a pre-requisite for the forms of social life under discussion, might be giving adherents of apriorism too much to play with.

Sidestepping the causal logistical aspects of this quandary, the problem might be at least partly ameliorated through recognition that the “cognitive capacity” in question could be an embodied one, and so not “a priori” in any traditionally cognitivist sense. Here it is worth remembering that, like Sohn-Rethel's “real abstraction,” Lakoff and Johnson's “embodied concepts” were likewise an attempt to surmount the apriorism/empiricism divide by grounding elements of conceptual thought in material being:

Reason is not, in any way, a transcendent feature of the universe or of disembodied mind. Instead it is shaped crucially by the peculiarities of our human bodies.

(Lakoff and Johnson, 1999, p. 4)

Perhaps a synthesis of Sohn-Rethel's and Lakoff and Johnson's insights might provide fruitful terrain for future research.

## Conflict of interest statement

The author declares that the research was conducted in the absence of any commercial or financial relationships that could be construed as a potential conflict of interest.
